# The sphere-in-contact model of carbon materials

**DOI:** 10.1007/s00894-015-2895-7

**Published:** 2016-01-20

**Authors:** Constantinos D. Zeinalipour-Yazdi, David P. Pullman, C. Richard A. Catlow

**Affiliations:** Kathleen Lonsdale Materials Chemistry, Department of Chemistry, University College London, London, WC1H 0AJ UK; Department of Chemistry and Biochemistry, San Diego State University, 5500 Campanile Drive, San Diego, CA 92182-1030 USA

**Keywords:** Fullerene, Graphene, Graphite, Carbon nanotube, Molecular modeling

## Abstract

A sphere-in-contact model is presented that is used to build physical models of carbon materials such as graphite, graphene, carbon nanotubes and fullerene. Unlike other molecular models, these models have correct scale and proportions because the carbon atoms are represented by their atomic radius, in contrast to the more commonly used space-fill models, where carbon atoms are represented by their van der Waals radii. Based on a survey taken among 65 undergraduate chemistry students and 28 PhD/postdoctoral students with a background in molecular modeling, we found misconceptions arising from incorrect visualization of the size and location of the electron density located in carbon materials. Based on analysis of the survey and on a conceptual basis we show that the sphere-in-contact model provides an improved molecular representation of the electron density of carbon materials compared to other molecular models commonly used in science textbooks (i.e., wire-frame, ball-and-stick, space-fill). We therefore suggest that its use in chemistry textbooks along with the ball-and-stick model would significantly enhance the visualization of molecular structures according to their electron density.

**Electronic supplementary material:**

The online version of this article (doi:10.1007/s00894-015-2895-7) contains supplementary material, which is available to authorized users.

**Graphical Abstract:**
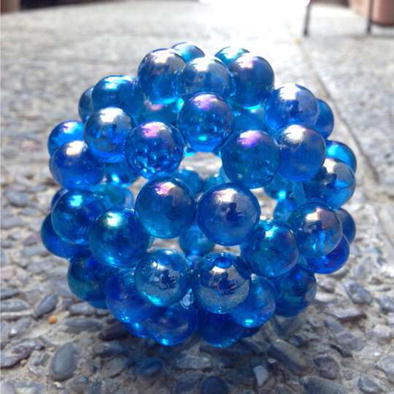
A sphere-in-contact model of C_60_-fullerene

## Introduction

Physical molecular and materials models have been used widely in chemistry teaching and research for about 100 years. The first such models consisted of spherical atoms connected via metallic bonds, in which the atoms were color-coded. These were introduced by the chemist August Wilhelm von Hofmann [1] in public lectures in 1860. Such models have nowadays been replaced mostly by the use of computer animations in molecular and material modeling where any structure can be designed either by entering the unit cell parameters or by building the molecules atom-by-atom (e.g., z-matrix). However it is evident that physical models of molecules and materials have been important not only in teaching but also in the discovery of new molecules and materials. A classic example is the structure of desoxyribonucleic acid (DNA), which was discovered by Watson and Crick [2] with the help of a 2-m high ball-and-stick model.

**Fig. 1:**
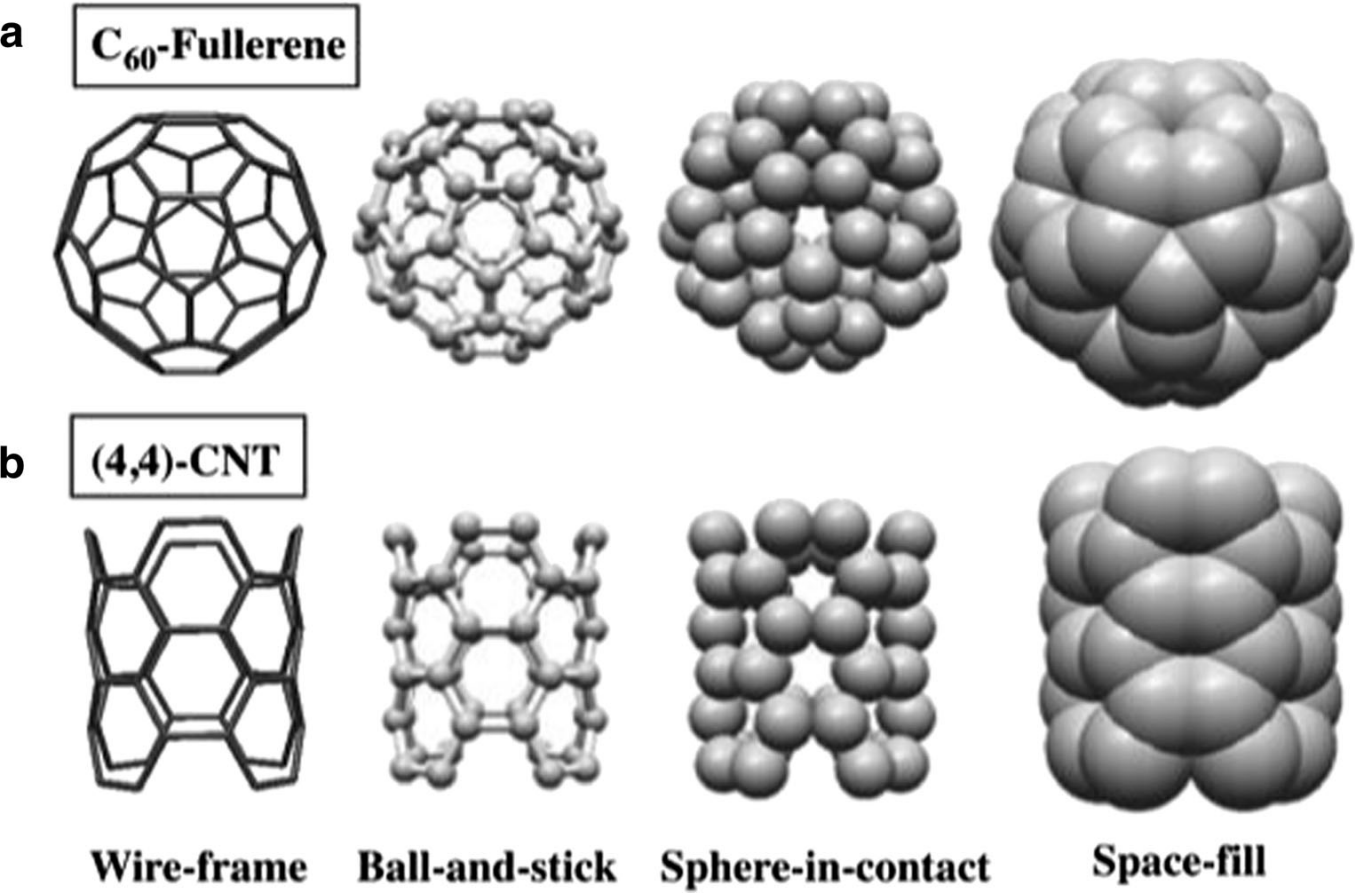
**a** C_60_-Fullerene and **b** (4,4)-carbon nanotube (CNT) in wireframe, ball-and-stick, sphere-in-contact and space-fill representations

Another example is the alpha-helix [3] that was discovered by Linus Pauling with the help of paper ribbon models, which showed the relative alignment of H-bonds in the helix [3]. In this paper we make a distinction between three commonly used models in physical molecular models, the *ball-and-stick*, *wire-frame* and *space-fill* models, and the *sphere-in-contact* model presented in Fig. 1 for C_60_-fullerene and (4,4)-carbon nanotube (CNT). In addition, there is the ribbon model, which is used to show the tertiary structure (a-helix, β-sheet) in biological molecules, which is found only in computer graphics software and is therefore not considered here.

The first molecular model kit that had the structure of molecules in the way they are taught in general chemistry courses was invented by the chemist Andre Dreiding in 1958, with wire-frame models for organic molecules. Even though Dreiding’s stereomodels show clearly the connectivity of the atoms, these representations lack information about the space occupied by the atoms. Therefore, in 1952, Corey and Pauling [4] introduced the space-fill models, which are among the most accurate representation of matter as they take into consideration that atoms occupy space. Here, bonded atoms are represented by overlapping spheres of radius that are close to the van der Waals (VdW) radius C_non-bonded_ = 1.5 Å, C_bonded_ = 1.25 Å) [4] rather than the atomic radius (AR) of each element, which results in bond lengths (BL) between atom A and B that are generally smaller than the sum of their VdW radii,

1 $$ BL<{r}_{A,VdW}+{r}_{B,VdW} $$

These models distinguish the main elements of organic compounds according to a color code (i.e.,* sky-blue* N,* white* H,* black* C,* red* O). This type of representation of molecular models is of particular use in supramolecular chemistry where molecules are bound through weak interactions (e.g., H-bonding, VdW), in that they will keep the interacting atoms at the correct separation in order to minimize their interaction potential. This is useful as many molecular properties rely on stereochemistry (e.g., supermolecular complexation, intramolecular rotation) and can be rationalized based on how atoms, which have VdW radius, can move in the void space that is present in molecular structures, while maintaining their chemical bonds.

In this paper we present the sphere-in-contact model, which can be used to construct molecular models of carbon materials (e.g., graphite, graphene, carbon nanotube, fullerene). A survey taken among chemistry and computational modeling students about the use and usefulness of this molecular model compared to other commonly used molecular models such as the ball-and-stick, wire-frame and space-fill models is then presented. Subsequently we show simplified schematics and describe a procedure of how one can build a sphere-in-contact model for fullerene.

## Materials and methods

Different materials have been used to build molecular models and the choice is usually based on expense, aesthetics and the practicality of handling the material. Sir William L. Bragg suggested 90 years ago how to use wax balls in the building of models of materials [5]. Cork balls [6] have also been used for molecular models in organic chemistry but since the broad use of synthetic plastics, plastic spheres connected initially by a metal wire [7] and subsequently by plastic deformable joints, have been the materials of choice for molecular model kits. There are also several papers that have described the use of various materials for the building of fullerenes, including plastic beads [8], bottle caps [9] and origami paper constructs [10]. Here, we used 1.4-cm transparent glass marbles and a 2-component (resin and hardener) epoxy resin adhesive to bind the marbles. Dried epoxy resin glue is relatively hazard and safety free when handled with care. Uncured epoxy resin ingredients [Bisphenol-A-(epichlorohydrin)] may cause sensitization of skin and irritation of the eyes. It is advisable to build these models in a well-ventilated space or outside wearing plastic gloves and closed safety glasses.

## Results and discussion

### Sphere-in-contact model of carbon materials

**Fig. 2:**
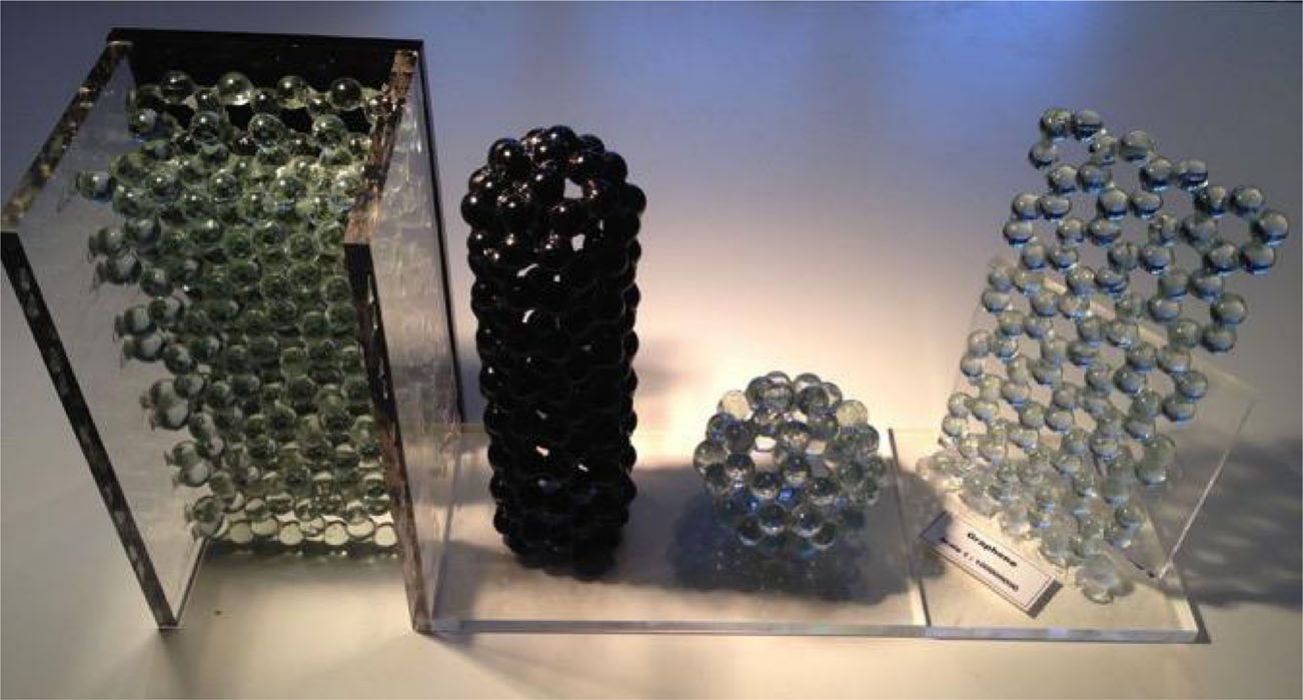
Sphere-in-contact model for graphite, CNT, C_60_-fullerene and graphene made out of marbles and epoxy glue. The scale of the models is 1:10^8^. The models are held in place using polymethyl methacrylate (PMMA) sheets of 1 cm in width that were bound to the models with epoxy glue. The PMMA sheets were bound together by solvent adhesive

Actual physical models of carbon materials that we have built are shown in Fig. 2. In particular we have used this model with success to construct models for graphite [11], graphene, capped CNT and fullerene, but one can essentially build any desired structure (e.g., carbon nanocones, curved graphene sheets) as long as a supporting structure (that can be removed with ease) is present until the adhesive has dried. For the atoms we used 1.4-cm diameter glass marbles so that the scale of the model is about 1:10^8^. One can also use inexpensive materials (e.g., acrylic plastic spheres); however, it was noted that there is an aesthetic advantage of models made of glass marbles, especially when transparent glass is used, as one can observe in each atom, different parts of the structure and the background, which generates a nice visual effect. It is therefore suggested that the base color of the structure of other materials is transparent so that, e.g., alloys and interstitial structures are readily visible. In Fig. 2 we present the sphere-in-contact model for graphite, a (4,4)-CNT with a cap, C_60_-fullerene and graphene. All models have the same scale, which is 1:10^8^, so that one centimeter measured on the model corresponds to 1 Å in the real structure. A definite advantage of building the structure of carbon materials with the sphere-in-contact model is that one can build these structures with very little input from trigonometric algebra, as the spheres when in contact are at almost the correct distance according to their bond length.

According to Bragg, to a first approximation, the BL between atoms is equal to the sum of their atomic radius (i.e., covalent radius) [12] given by

2 $$ BL\approx {r}_{A,AR}+{r}_{B,AR} $$

We note that, in the sphere-in-contact model, one can see the space where most of the electron density is located (>98 %), which yields a better representation of the void space present in the structure. In the sphere-in-contact model of graphite, this was particularly useful when explaining the properties as the model shows that the distance between the layers is more than two times the distance between adjacent carbon atoms within each sheet—a property associated to the difference in intra-layer (covalent) and inter-layer (VdW) interactions. We could also easily explain the differences between the stacking of the layers in hexagonal (ABAB) and rhombohedral (ABCA) graphite. Additionally, we noticed that regular diffusion through the layer is unlikely, except for elements with very small atomic radius (e.g., helium [13]), but diffusion between the layers (e.g., lithium-cation [14]) is possible as there is about 1.7 Å of void space between two consecutive layers of graphite. The space is sufficient to fit small molecules such as water (with its molecular axis lying along the basal plane), which may explain the exfoliation of graphene from graphite in water [15]. So it is evident that such models are useful teaching supplements but also helpful in the discovery of new materials or in the rationalization of their physical properties.

### Survey about molecular models in chemistry

**Fig. 3:**
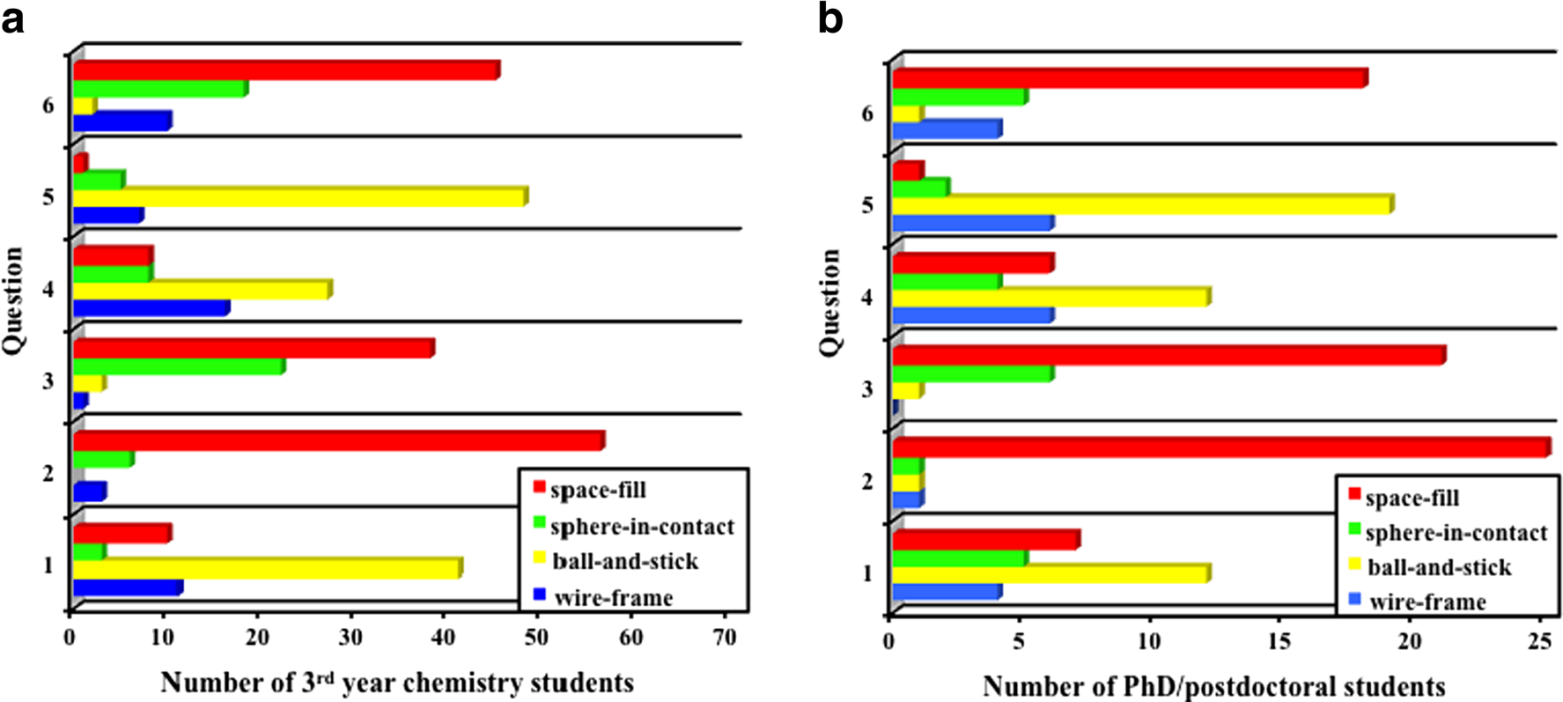
*Bar diagram* of the number of **a** 3rd year chemistry undergraduate students, and **b** PhD/postdoctoral students with a computational modelling background that marked answers* A*,* B*,* C* and* D* in the survey questions, respectively. Note that for Q6, 15 % of the students gave two answers, which were both taken into consideration for the survey analysis. A copy of the survey questionnaire is given as supporting information in Fig. S1

The model presented here, the sphere-in-contact model, is an intermediate between the ball-and-stick model and the space-fill model, and it is conceivable that it will have certain advantages and disadvantages compared to other commonly used molecular models. We assessed the usefulness of this model via a survey that was taken on a voluntary basis by about 100 participants with a university-level chemistry and computational modeling background. In the survey, the participants were asked to choose among the four molecular models (i.e., the sphere-in-contact, wire-frame, ball-and-stick, and space-fill) with respect to whether they represent the properties (e.g., electron density) of common carbon materials in an improved approach. The survey was analyzed and certain conclusions are tentatively reported in the following section. In particular, the survey was based on the answers given on a voluntary basis by 3rd year physical chemistry students (G1), and PhD and postdoctoral students (G2) with a computational modelling background during the 1st week of classes in 2015 at an acclaimed University in London. This questionnaire was composed of six multiple-choice questions, of which three referred to Fig. 1. Each participant was offered anywhere between 5 min and 30 min time after which the survey was collected. Only 10 % of the participants turned in a blank survey and, of the remaining 90 %, G1 answered all questions whereas G2 did not answer some of the questions and in some cases gave two answers. There was a total of 65 undegraduate and 28 PhD/postdoctoral participants, which were analyzed in two separate groups, G1 and G2, respectively. The survey bar diagram and the percentage of each answer are shown in Fig. 3 and Table 1, respectively.

**Table 1:** Percentages of each answer given in the survey. The survey questions are given in the text and in Fig. S1

Question	A%	B%	C%	D%
Number of 3rd year chemistry undergraduates = 65				
1	17	63	5	15
2	5	0	9	86
3	2	5	34	58
4	25	42	12	12
5	11	74	8	2
6	15	3	28	69
Number of PhD and postdoctoral students = 28				
1	14	43	18	25
2	4	4	4	89
3	0	4	21	75
4	21	43	14	21
5	21	68	7	4
6	14	4	18	64

In Question 1 (Q1), the subjects were given Fig. 1a and the following question, “*Which of the following models do you think is a more accurate representation of the actual structure of fullerene C*_*60*_*?*” The answer we were anticipating was the sphere-in-contact model as there the atoms enclose most of the electron density and the connectivity between (contact point between spheres) the atoms can still be realized. However, we also considered the space-fill model to be correct on the basis that it encloses all the electron density and shows the surface of minimum approach, if another molecule is interacting with the carbon material. To our surprise most of the participants entered this answer incorrectly. Here, G1 choose first ball-and-stick, followed by wire-frame, whereas G2 chose ball-and-stick followed by space-fill. So only a combined 20 % of G1 and 43 % of G2 answered correctly in this questionnaire, which clearly suggests that one in five undergraduate students do not have a correct understanding of where the electron density is in carbon materials, a ratio that becomes one in two for PhD and postdoctoral students. It is therefore evident that the sphere-in-contact model would significantly enhance the students’ perception if explained on a conceptual basis, as being the structure that shows where most of the electron density is.

In Q2 “*In which model of C*_*60*_*is the connectivity of the atoms not readily visible ?*” the results are almost identical for undergraduate and PhD/postdoctoral students. Here, 86 % of G1 and 89 % of G2 answered correctly that the space-fill model does not readily show the connectivity between the atoms.

In Q3 they were given Fig. 1b and the following question, “*Which model shows the volume where most of the electron density is ?*” Here the correct answer was the sphere-in-contact model as a sphere of atomic radius encloses most of the electron density of carbon. The electron density outside this sphere, such as the electron density that belongs to chemical bonds and the diffuse electron density that corresponds to the tails of the atomic wavefunctions, is very small (<1–2 %). However, here the question was possibly read as “*Which model shows where most of the electron density is ?*” and therefore 58 % of G1 and 75 % of G2 chose the space-fill model. So the survey results here indicate that it would be useful on a conceptual basis to also learn about the sphere-in-contact model as it shows where most of the electron density is but simultaneously shows the connectivity between the atoms (e.g., lattice).

In Q4 they were given Fig. 1b and the following question, “*Which of the following models do you think is a more accurate representation of the actual structure of a (4,4) carbon nanotube (CNT) ?*” This was a control question for Q1 to see if the participants were answering some of the questions randomly or if their answers were based on their understanding of the three-dimensional (3D) structure of molecular structures. If there was randomness in their answers then the relative percentages for each question would be different when compared to Q1. Here the results for G1 and G2 were 61 % and 85 %, respectively, consistent with the answer given in Q1. This clearly shows that the answers given were not random and they were based on the participants’ understanding of molecular models.

In Q5 “*Which is the most commonly used molecular model in general chemistry textbooks ?*” the correct answer was the ball-and-stick model, which 74 % of G1 and 68 % of G2 answered correctly.

In Q6 “*Which is the least commonly used molecular model in general chemistry textbooks ?*” the results were that 69 % of G1 and 64 % of G2 answered the space-fill model. However, the correct answer was the sphere-in-contact model, with the space-fill being second least common. Possibly the similarities of the sphere-in-contact model and the ball-and-stick model confused the participants choice in this question as they answered incorrectly that they had seen the sphere-in-contact model in science textbooks more frequently, something that could not be confirmed in ten general and physical chemistry textbooks used by Universities world-wide (a list of these textbooks is given as supporting information Fig. S2).

For carbon materials (e.g., graphene, graphite, carbon nanotubes, fullerenes, carbon nanocones) space-fill models according to our survey are not generally used in chemistry textbooks. This is perhaps because the bonding framework (e.g., the hexagonal lattice in graphite) is not readily visible since the void space (space where electron density if very small) is significantly underestimated, especially when VdW radius is used for the atoms *r*_C,VdW_ = 1.7 Å). However, in the sphere-in-contact model, the void space is described accurately, because the atoms are modeled by spheres of atomic radius *r*_C,AR_ = 0.7 Å). We therefore note that the concept of void space is one that ought to be built in molecular models as it offers stereochemical information, based on which one can estimate whether atoms/ions can diffuse in a material, if radiation can penetrate as the void space would not scatter it significantly and many other properties that rely on the electron density of molecules and materials.

It is apparent from our survey analysis that the sphere-in-contact model in combination with the more commonly used wire-frame or ball-and-stick model would significantly improve the visualization of 3D structures of molecules in terms of their electron density.

### Sphere-in-contact model of fullerene

**Fig. 4:**
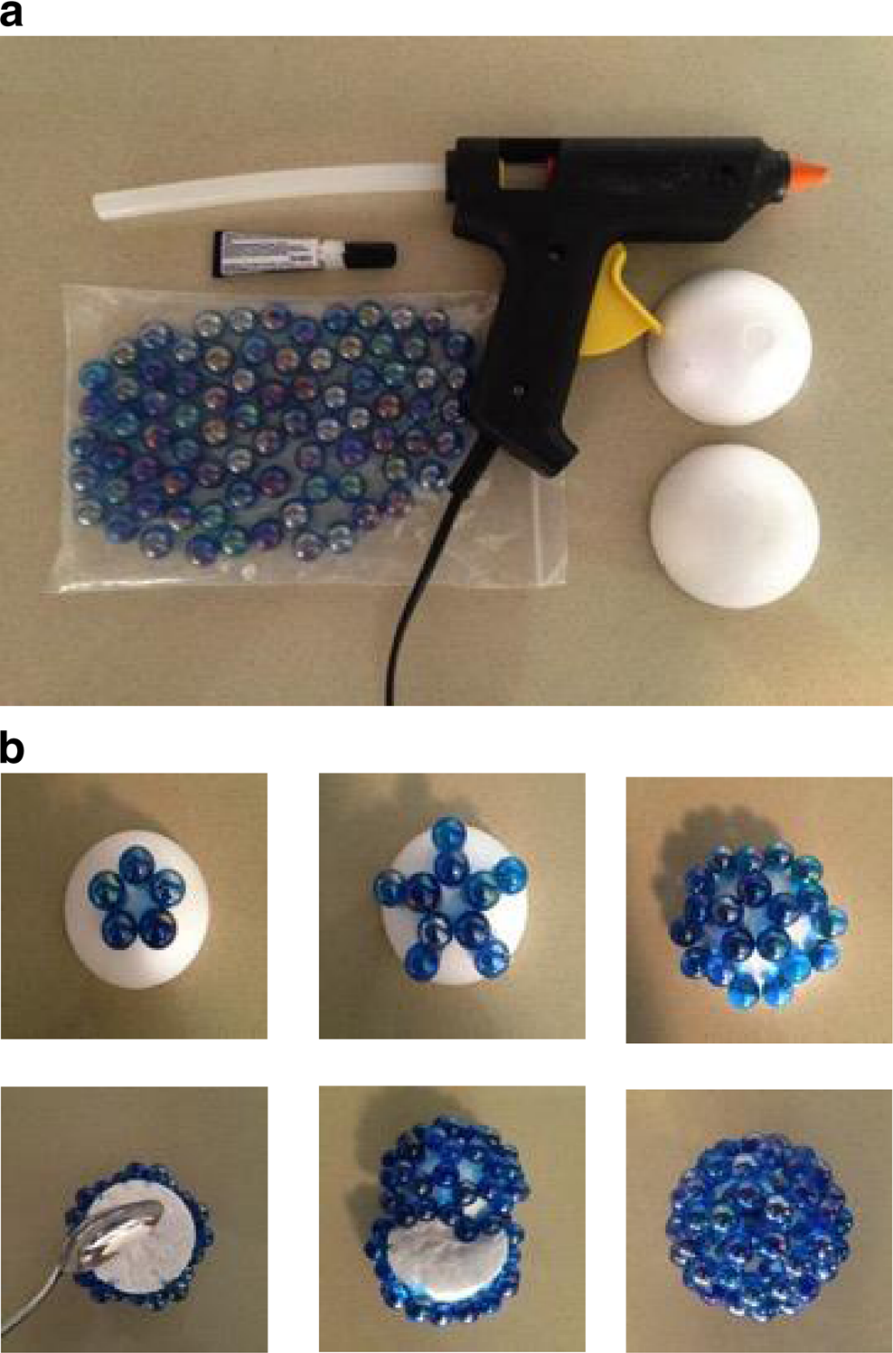
**a** Materials and tools (i.e., hot-glue ‘gun’, styrofoam sphere ∅ = 5.3 cm, 60 × 1.4 cm marbles, super or epoxy glue) needed to build a model of C_60_-fullerene; and **b** a pictorial procedure for the construction. Note that the five-fold symmetry axis is perpendicular to the base of the styrofoam hemisphere

In the following section we describe the procedure to construct a sphere-in-contact model C_60_-fullerene, which costs only £3 so could be constructed readily in any workshop. These models can be used in a University environment as a visual aid to teaching. We have developed a procedure in which two hemi-spheres of C_60_ are built, which are then brought together. The materials required are shown in Fig. 4a and comprise a hot-glue ‘gun’ for initially binding weakly the marbles to the styrofoam, 2 styrofoam hemispheres and 60 × 1.4-cm spherical marbles.

**Fig. 5:**
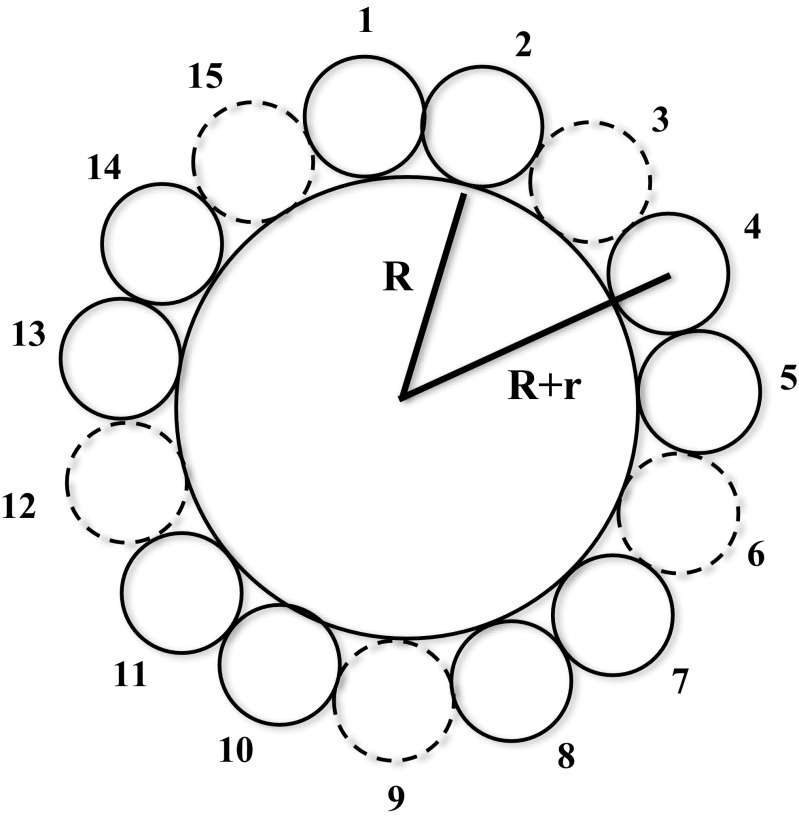
Simplified schematic showing the cross-section of C_60_-fullerene using a sphere-in-contact design

The diameter of the styrofoam can be approximately calculated via the following trigonmetric equation,

3 $$ R\cong \frac{15r}{\pi }-r, $$

where R and r are the radius of the styrofoam and marbles, respectively. This equation was derived based on the cross section of C_60_ shown in Fig. 5. The sphere, which has its surface on the centers of the 15 marbles, had a perimeter of about 15 × 2 × r. Since the perimeter of this circle is also given by 2π(*R* + r), equating these two relationships yields Eq. 3.

The marbles are attached via the hot-glue ‘gun’ starting from the top pentagon, adding five hexagons and then five pentagons around it, in the procedure pictured in Fig. 4b. Once the marbles are in place they can be permanently bound by mixing epoxy glue and hardener thoroughly and by placing a small amount at the contact point between every two marbles. The epoxy glue starts to cure after 1–2 min although some time should be allowed, and then the Styrofoam can be removed with the use of a large spoon. The last step is to bring the two fullerene hemispheres together via the help of adhesive tape and use epoxy resin again to permanently bind them. We note that this procedure can be developed into a 2-h workshop for chemistry students.

## Conclusions

A sphere-in-contact model is presented and applied to build the structure of various carbon materials (i.e., graphite, graphene, carbon nanotubes and fullerene). A detailed procedure is presented for fullerene with which one can build inexpensive models of this material. These molecular models have scale in contrast to other molecular models (i.e., ball-and-stick, wire-frame and space-fill) where scale is not always a characteristic. Furthermore, the model has the correct proportions for the atoms based on their electron density. Based on a survey taken among 65 undergraduate chemistry students and 28 PhD/postdoctoral students with a background in molecular modeling we found that there are misconceptions that arise from the incorrect visualization of the size and the location of the electron density in such materials. We provide evidence on a conceptual basis that the sphere-in-contact model has improved molecular representation of the electron density of carbon materials. We therefore suggest that it could be used in teaching and research where the visualization of molecular structure according to the electron density is important such as in the design of novel doped materials.

## Electronic supplementary material

Supporting information file contains the survey questionnaire (Fig. S1) and a list of chemistry textbooks that were considered in this study (Fig. S2).

ESM 1 (PDF 440 kb)
