# Supplementary material for: The sphere-in-contact model of carbon materials
Source: J Mol Model. 2016 Jan 20;22:40. doi: 10.1007/s00894-015-2895-7 (PMC4720691; doi:10.1007/s00894-015-2895-7)
Supplement: Supplementary file 1 — (PDF 440 kb) [file 894_2015_2895_MOESM1_ESM.pdf]

Supporting Information

The Sphere-in-Contact Model of Carbon Materials

Constantinos D. Zeinalipour-Yazdi[1,*], David P Pullman[2], C. Richard A. Catlow[1]

[1]Kathleen Lonsdale Materials Chemistry, Department of Chemistry, University College London, London, WC1H 0AJ, UK

[2]San Diego State University, Department of Chemistry and Biochemistry, 5500 Campanile Drive, San Diego, CA 92182-1030, USA

Date here *contact info: uccazei@ucl.ac.uk*

Model of carbon materials questionaire

Please circle one:

1. Which of the following models do you think is a more accurate representation of the actual structure of Fullerene C_{60}?

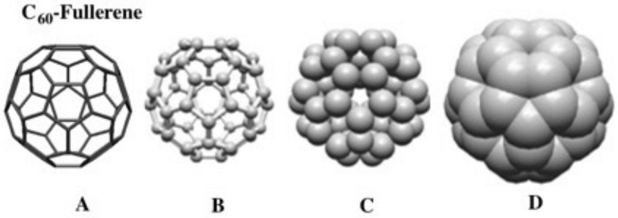

2. In which models of C_{60} is the connectivity of the atoms not readily visible ?

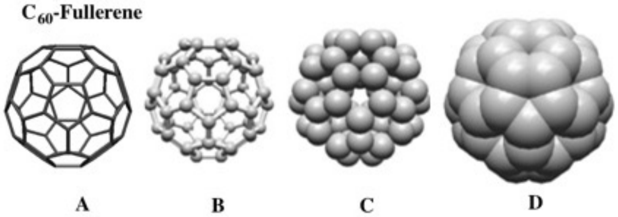

3. Which model shows the volume where most of the electron density is ?

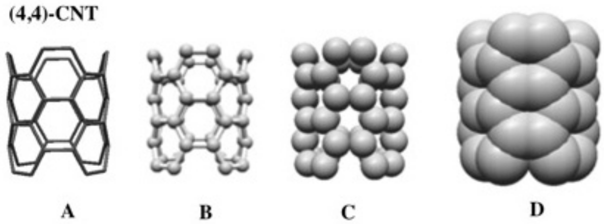

4. Which of the following models do you think is a more accurate representation of the actual structure of a 4×4 carbon nanotube (CNT) ?

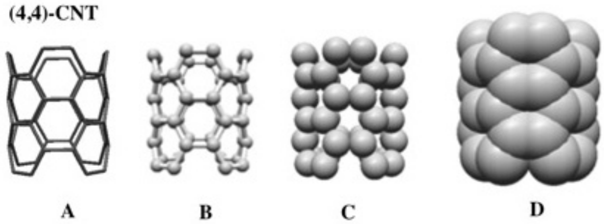

5. Which is the most commonly used molecular model in general chemistry textbooks ? **A** **B** **C** **D**

6. Which is the least commonly used molecular models in general chemistry textbooks ? **A** **B** **C** **D**

S-Figure 1. Survey questionnaire given to **G1** and **G2**

1. General Chemistry, Darell D. Ebbin and Steven D. Gammon, 6th edition, 1999.
2. Chemistry, The Molecular Nature of Matter and Change, Martin S. Silberberg, 3rd edition, McGraw Hill, 2003.
3. General Chemistry, The Essential Concepts, Raymond Chang, Annotated Instructor's Edition, 4th edition, Mc Graw Hill, 2006.
4. Introductory Chemistry, Steve Russo and Mike Silver, 3rd edition, Pearson, 2007.
5. General, Organic and Biological Chemistry, Structures of Life, Karen C. Timberlake, Platinum edition, Pearson, 2004.
6. Chemistry, Matter and Its Changes, James E. Brady and Fred Senese, 4th edition, Wiley, 2004.
7. The Practice of Chemistry, Donald J. Wink, Sharon Fetzer-Gislason, Sheila D. McNicho-las,W.H. Freeman and Company, 2004.
8. Chemistry in your Life, Colin Baird, 2nd edition, W.H. Freeman and Company, 2006.
9. Physical Chemistry for the Life Sciences, Peter Atkins and Julio de Paula, Oxford University-Press and W.H. Freeman and Company, 2006.
10. General Chemistry, Linus Pauling, Dover Publications Inc., 1970.

S-Figure 2. Titles of various undergraduate chemistry textbooks that have been checked whether they have the sphere-in-contact model.
